# Safe return to driving after severe acquired brain injury: toward a tiered multidisciplinary rehabilitation pathway

**DOI:** 10.3389/fneur.2026.1751388

**Published:** 2026-03-23

**Authors:** Donatella Saviola, Stefania Bruni, Matteo Cantoni, Antonio De Tanti

**Affiliations:** Centro Cardinal Ferrari KOS, Fontanellato, Parma, Italy

**Keywords:** driving rehabilitation, driving simulator, fitness to drive, multidisciplinary approach, severe acquired brain injury, return to driving

## Introduction

Driving is among the most demanding instrumental activities of daily living requiring continuous integration of perception, cognition, motor control, and behavioral regulation under real-time conditions (1). For people recovering from severe acquired brain injury (sABI), driving symbolizes regained autonomy, social integration, and reduced family dependence (1), and is linked to return to employment (2).

Michon's hierarchical model of driving control remains a useful framework to understand why brain injury threatens driving safety (3). Damage to frontal and parietal networks may disrupt strategic planning, such as route choice and risk evaluation, as well as tactical decision-making, including lane positioning and speed adjustment. Even when gross motor recovery is achieved, subtle impairments in attention, spatial exploration, executive control, or emotional regulation can significantly affect driving performance (4).

Empirical studies show that resuming driving enhances social engagement, functional performance, mood, and life satisfaction (5). Yet post-injury driving management varies widely (1), as many regulations focus primarily on medical eligibility or legal “fitness-to-drive” criteria (e.g., seizure-free intervals, minimum visual fields) that, while necessary, do not adequately reflect functional driving competence. A major barrier is system-level fragmentation: neurologists, neuropsychologists, occupational therapists, and rehabilitation specialists often work separately, without a unified framework linking assessment, rehabilitation, and licensing. This can lead to both premature exclusion and unsafe return to driving.

Taken together, these limitations highlight the need to reconceptualize post-ABI driving as a structured rehabilitation process rather than a one-time fitness-to-drive examination. A Tiered Multidisciplinary Rehabilitation Pathway can integrate medical, functional, and legal dimensions within a coherent, equitable, and evidence-based system. The proposed model draws on clinical experience at the Centro Cardinal Ferrari hospital and international evidence from the neurorehabilitation and occupational therapy literature.

## Current gaps in practice

Current practice remains highly fragmented. Many patients are referred for driving assessment only at the end of general neurorehabilitation. At the same time, licensing authorities frequently rely on static measures such as visual acuity, seizure history, or motor deficits, despite evidence that neuropsychological performance, visual scanning behavior, and executive self-monitoring correlate more strongly with real on-road outcomes (4, 6). This mismatch creates ethically problematic situations: some individuals return to driving without adequate rehabilitation, while others are restricted despite having compensable or trainable deficits. Fragmentation across disciplines (neurology, physiatry, neuropsychology, ophthalmology, physiotherapy, occupational therapy, and driving instruction) further limits coherent decision-making, as services often operate in parallel rather than within an integrated pathway.

## Advances across rehabilitation domains

### Neuropsychological domain

Safe driving depends on attention, working memory, executive function, processing speed, and visuospatial abilities, domains frequently affected after sABI. Importantly, impairments may persist even when global screening tests appear within normal limits, and specific deficits in divided attention, executive control, visuo-spatial organization, and visual processing speed are among the strongest predictors of on-road failure (4, 7, 8). Targeted neuropsychological rehabilitation increasingly uses computerized exercises to train sustained and divided attention, working memory, and visuospatial processing. Platforms like Cogniplus (9) or Vienna Test (10) provide structured, measurable exercises that complement simulator-based training and facilitate transfer of cognitive improvements to real-world driving performance. These tools allow clinicians to monitor progress quantitatively and adjust therapy intensity over time.

Collateral information from relatives is also essential, as it can reveal real-world functioning and uncover inefficiency, inertia, impulsivity, poor adherence to routines, such as sleep patterns, medication, or substance use, which patients may underestimate or fail to report. This perspective is important because unreported risk-taking behaviors or disinhibition can undermine driving safety even when formal cognitive scores appear adequate (11, 12).

A further crucial issue is impaired self-awareness: individuals with frontal or right-hemisphere lesions may underestimate their deficits, exhibiting overconfidence and unsafe behaviors (13). For this reason, rehabilitation should integrate metacognitive and risk-management interventions addressing insight, behavior, medication, fatigue, and alcohol use. Studies suggest that combining neuropsychological findings with simulator-based training enhances the ecological validity of training and supports safer return-to-driving decisions (14, 15).

### Visual and ophthalmologic domain

Although vision is fundamental to safe driving, licensing standards still rely mainly on visual acuity, a parameter that only partially reflects functional performance. Individuals may meet legal thresholds yet present with hemianopia, quadrantanopia, neglect, impaired oculomotor control, diplopia, or slowed visual processing, all of which compromise hazard detection, spatial judgment, and reaction time (8, 16). A functional vision assessment should therefore evaluate visual fields, fixation, scanning efficiency, vergence, ocular alignment, and oculomotor coordination under conditions that approximate real-world demands.

Targeted visual rehabilitation can meaningfully support driving readiness. Biofeedback-based technologies, such as the Retimax Vision Trainer, may represent a restorative approach when effective, using visual and auditory feedback to improve fixation stability and visual scanning behavior. Preliminary studies suggest that this approach can enhance visual exploration in individuals with sABI, potentially supporting safer visual behavior during driving tasks (17). By contrast, compensatory scanning programs, prism adaptation, and targeted interventions for diplopia or field-loss compensation aim primarily to foster compensatory strategies. Taken together, these approaches suggest that visual rehabilitation can contribute to functional driving recovery (18).

However, regulatory practices remain inconsistent: some authorities accept compensatory or adaptive mechanisms, others apply categorical restrictions. A shift toward functional and contextual evaluation of vision, rather than reliance on static parameters such as acuity, is essential for fair and evidence-based decision-making in post-ABI driving rehabilitation.

### Motor and physiotherapy domain

Motor impairments following sABI may compromise steering control, pedal coordination, and reaction time. Deficits in trunk stability and limb coordination are directly associated with increased accident risk (19). Rehabilitation programs should therefore include driving-specific physical training, focusing on bilateral lower-limb transitions, trunk rotation, and endurance.

Where residual impairments persist, adaptive devices such as hand controls, pedal extensions, and steering aids may permit safe participation (15). Combined physiotherapy and occupational therapy interventions integrating adaptive technology have been shown to improve both performance and self-efficacy.

### Occupational therapy and functional integration

Occupational therapy bridges impairment-level assessments and real-world performance. On-road testing in dual-control vehicles remains the gold standard. Still, it should be embedded within a graded rehabilitation pathway that includes simulator exposure, fatigue management, and adaptive equipment training (20).

OT also supports families, often jointly with the neuropsychologist/psychologist, by addressing the emotional impact of driving restriction, facilitating shared decision-making, and ensuring transparent, patient-centered communication.

### Simulation and technology

Driving simulators and virtual reality platforms offer ecologically valid environments to assess how cognitive, visual, and motor domains interact under real-time demands. Variables such as lane deviation, hazard anticipation, and braking latency correlate with on-road performance (21, 22). Simulation also supports rehabilitation by allowing patients to rehearse strategies and improve self-awareness before real traffic exposure.

While simulators vary in fidelity and may induce cybersickness, particularly when immersive virtual-reality systems are used, their controlled environment enhances safety and reduces anxiety. Evidence suggests that simulator-based training improves the predictive accuracy of licensing decisions and facilitates the transition to on-road evaluation (15).

## Toward a tiered multidisciplinary rehabilitation pathway

When considered together, these domains support the need for a structured, tiered approach to post-injury driving rehabilitation. Legal prerequisites define only a baseline for eligibility: in Italy, for example, a 12-month seizure-free interval and a minimum binocular visual field of 120 degrees. Regulations also allow adaptive vehicle modifications and restricted licenses, which regional commissions use as individualized risk-management tools balancing autonomy and safety.

However, these requirements alone are insufficient to determine true fitness to drive. Longitudinal data show that well-controlled post-traumatic or post-anoxic epilepsy does not preclude safe driving (23), underscoring the need for multidisciplinary evaluation rather than categorical exclusion based solely on diagnosis. Behavioral and lifestyle factors, including alcohol or substance misuse, further influence licensing decisions and are handled differently across regions, with some relying on objective testing, such as hair analysis, or structured rehabilitation programs before granting or reinstating driving privileges, particularly when post-injury screening is positive or clinical suspicion emerges after a brain injury. Given that individuals with sABI may underestimate such risks, systematic follow-up assessments are essential. These risks are well-documented in the literature, which shows that post-sABI individuals may underestimate or underreport substance use and impulsive behaviors that significantly increase crash probability (24–26).

**Figure 1:**
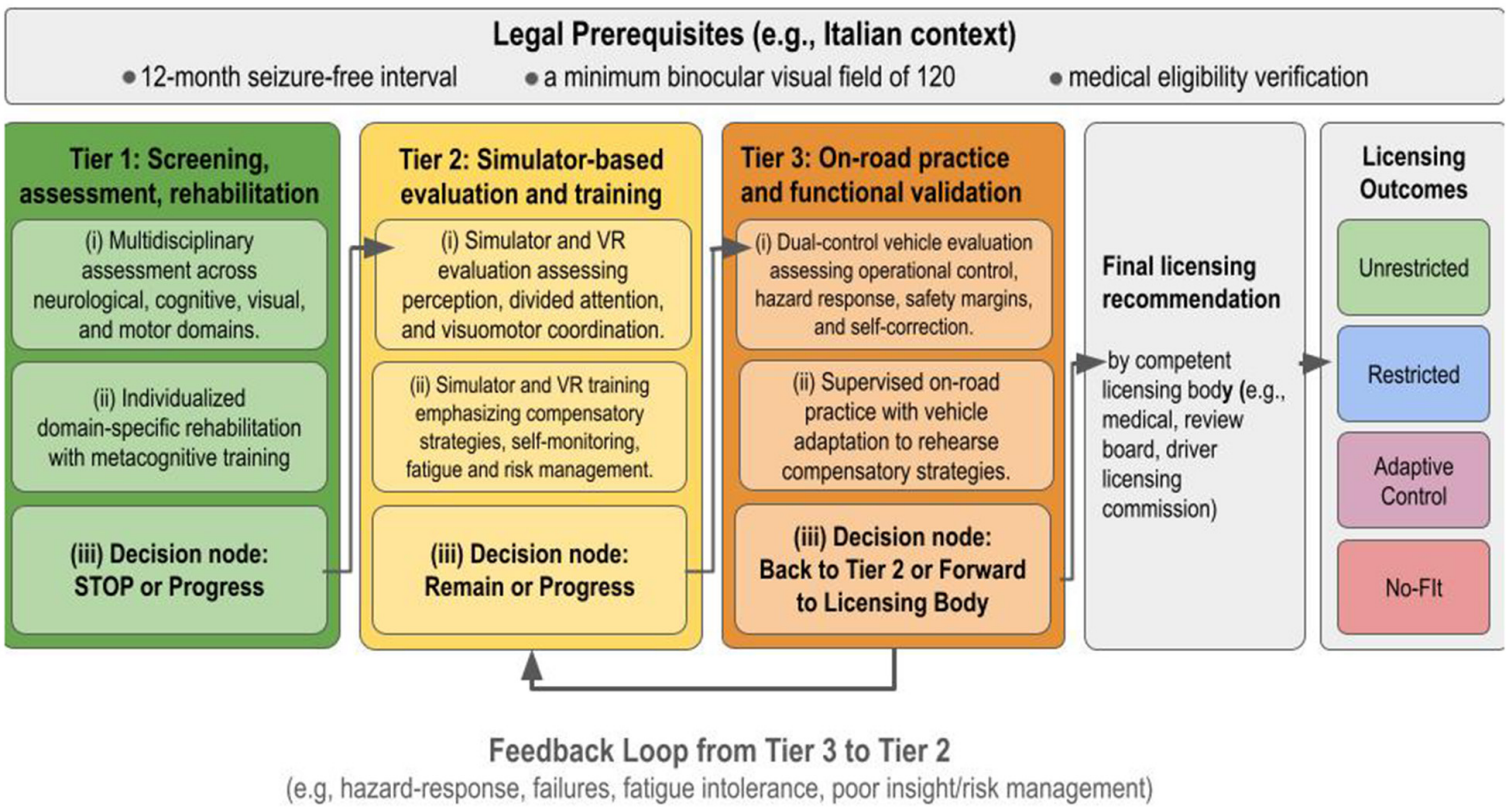
Tiered, iterative rehabilitation pathway for return to driving after severe acquired brain injury (sABI). Each tier includes (i) tier-specific assessment, (ii) targeted rehabilitation/training, and (iii) a decision node (progress/remain/stop). A feedback loop redirects patients from Tier 3 to Tier 2 when on-road findings indicate modifiable deficits (e.g., hazard-response failures, insufficient compensatory scanning, fatigue intolerance, poor insight/risk regulation, or need for adaptive controls training), and patients may remain in Tier 2 until stable, criterion-level performance is achieved. Final licensing recommendations are made by the competent licensing body, such as a medical review board or driver-licensing commission, based on integrated evidence across tiers, with on-road evaluation providing functional validation.

We therefore propose a three-tier rehabilitation pathway (see Figure 1) that explicitly links assessment to targeted intervention and defines decision points, responsibilities, and outcomes.

### Operational definition of the tiered rehabilitation pathway

We use the term “rehabilitation pathway” to denote a structured, stepwise, multidisciplinary process that links assessment to targeted intervention and culminates in a documented licensing recommendation. Across tiers, the workflow is: (i) screening and eligibility checks, (ii) multidisciplinary assessment, (iii) individualized rehabilitation/training addressing identified impairments, (iv) re-assessment to verify change, and (v) a decision node (progress/repeat/stop) with explicit outputs. Roles are defined per domains, and timing is anchored to clinical milestones (medical stability, outpatient readiness, and consolidation of compensatory strategies) rather than fixed intervals, to accommodate heterogeneity in recovery trajectories after sABI.

#### Tier 1—Screening, multidisciplinary assessment, and early targeted rehabilitation

The first tier combines baseline evaluation across neurological, cognitive–metacognitive, visual, and motor domains with the early initiation of individualized rehabilitation. Assessment establishes medical stability and driving-relevant prerequisites (e.g., seizure control and treatment adherence), characterizes attention and executive functions together with insight and risk regulation, evaluates visual fields/oculomotor function and neglect, and documents motor control relevant to vehicle operation. Rehabilitation targets modifiable deficits through domain-specific interventions and metacognitive work focused on self-awareness and safety-related decision-making. The Tier 1 decision point is provisional: patients proceed to Tier 2, repeat Tier 1 with adjusted goals, or stop when safety-critical impairments are non-remediable. Indicative timing is anchored to rehabilitation milestones rather than fixed intervals.

#### Tier 2—Simulator-based functional evaluation and enhanced training

The second tier builds on this foundation through simulator-based evaluation and enhanced training, including virtual or simulator environments where available that allow patients to practice hazard perception, divided attention, and visuomotor coordination in progressively more demanding scenarios. Tier 2 also provides quantitative performance signatures (e.g., error patterns and reaction profiles) that clarify how domain-specific deficits manifest under integrated driving demands. Training is structured and progressive, emphasizing compensatory strategies, self-monitoring, fatigue management, and behavioral risk reduction. Progression to Tier 3 requires stable performance across sessions, demonstrable compensatory strategy use, and adequate self-awareness. Patients with residual but potentially modifiable deficits remain in Tier 2 or undergo targeted, domain-specific rehabilitation within the same multidisciplinary program before re-testing.

#### Tier 3—Supervised on-road practice and final functional validation

The third tier consists of on-road practice in dual-control vehicles, incorporating task-specific adaptation to the vehicle when necessary, so that patients can rehearse compensatory strategies and consolidate motor routines required for safe driving. Tier 3 provides the highest ecological validity by evaluating operational/tactical control, hazard response, safety margins, and the capacity to self-correct in real traffic. Nevertheless, licensing recommendations are not based on on-road performance in isolation. Finally, Tier 1 is led by rehabilitation physician/neurology with neuropsychology, OT/PT, and vision specialists as indicated; Tier 2 primarily by OT, PT, and neuropsychology with driving specialist support; Tier 3 by certified driving instructor/driving rehab specialist with OT and medical oversight.

#### Integration rule and outcomes

Final recommendations are derived from integrated evidence across tiers and documented through a structured multidisciplinary review. Medical risk factors, cognitive–metacognitive profile (including insight), visual function, motor/adaptive needs, simulator-derived patterns, and on-road behavior are synthesized to determine the safest outcome: unrestricted driving, restrictions (e.g., context/time limits), adaptive licensing, conditional approval with follow-up, continued rehabilitation with re-testing, or non-fitness when safety-critical deficits persist. The resulting documentation is forwarded to the competent licensing body (e.g., a medical review board or driver-licensing commission), which makes the final licensing decision.

#### Iterative structure

The pathway is intentionally iterative, with conditional repetition within Tier 2 and a Tier 3-to-Tier 2 feedback loop when on-road findings indicate modifiable deficits. This design ensures that progression reflects functional readiness rather than mere passage through stages.

## Discussion

Despite growing evidence and technological advances, comprehensive driving rehabilitation after sABI remains limited. Standardized assessment batteries and uniform regulatory interpretation are still lacking. European directives, most notably through Directive 2006/126/EC and subsequent updates, offer a coherent framework, but implementation is inconsistent (27, 28, 30). Access to driving simulators, adaptive equipment, and interdisciplinary teams is often restricted to larger centers, perpetuating geographic inequities. Moreover, longitudinal studies evaluating the impact of integrated rehabilitation on outcomes such as crash risk, psychosocial adjustment, or cost-effectiveness remain scarce (6).

The contrast between the availability of European-level norms and their inconsistent local adoption underscores the need for operational models that help clinicians translate regulatory principles into practice. A tiered multidisciplinary pathway can meet this need by integrating assessment, rehabilitation, functional recovery, self-awareness, adaptive behavior, and policy within a unified and clinically actionable framework.

## Clinical and policy implications

Adopting a structured pathway offers clinical, ethical, and policy advantages. It aligns rehabilitation with real-life priorities and reduces the risk of late-stage failure during licensing examinations. Neurological evaluation clarifies seizure risk, fatigue patterns, and medication effects. Motor assessment evaluates strength, coordination, postural control, and the motor efficiency of the locomotor apparatus. Neuropsychological evaluation encompasses attention, executive processes, processing speed and reaction time, visual processing, and visuo-spatial skills. Visual and ophthalmologic assessment considers fields, ocular motility, acuity, diplopia, quadrantanopia, and scanning efficiency, providing a realistic appraisal of driving readiness.

Technology enhances the process by offering effective training and performance data, while simulation and on-road evaluation remain essential to observe real-world performance and refine training strategies. Ethically, it replaces arbitrary decisions with transparent, evidence-based criteria that balance autonomy with public safety. From a policy standpoint, it provides licensing authorities with a structured dossier that combines data from all the above-mentioned domains, facilitating defensible licensing decisions.

Recognition of restricted or adaptive licensing as legitimate outcomes of structured rehabilitation would mark an important cultural and regulatory shift. It would acknowledge that safety and autonomy are not mutually exclusive but can coexist within a risk-managed, patient-centered framework. Such recognition would also promote equity by allowing individuals with residual but compensated deficits to maintain mobility under monitored conditions.

## Future directions and conclusion

Future progress requires multicenter validation of assessment batteries and simulator metrics, as well as registries enabling longitudinal tracking. Research should explore biomarkers, eye-tracking analytics, and the cost-effectiveness of tiered pathways. Within an ICF-informed view, the pathway links body functions (e.g., vision, cognition, motor control), activities (driving-related tasks), participation (mobility, work), and contextual factors (environmental and personal) to support transparent, multidisciplinary decisions (29).

Driving after sABI should be conceptualized as a continuum of rehabilitation, not a single evaluative act. A tiered, technology-supported, multidisciplinary pathway can promote safer and more transparent practice, balancing the right to mobility with public safety.
